# EUPopLink Country report - Romania

**DOI:** 10.12688/openreseurope.23593.1

**Published:** 2026-05-14

**Authors:** Elena Negrea-Busuioc, Andreea Stancea

**Affiliations:** 1 Department of Communication, National University of Political Studies and Public Administration, Bucharest, Romania

**Keywords:** populism, euroscepticism, Romania

## Abstract

This country report examines the evolving relationship between populism and Euroscepticism in Romania. While Romania has traditionally maintained strong public support for European Union membership, recent political developments reveal emerging tensions beneath this pro-European consensus. The report highlights a paradox: widespread endorsement of EU and NATO membership coexists with growing sovereigntist and anti-globalist sentiments. The rise of populist actors, particularly AUR, SOS Romania, POT, and the independent figure Călin Georgescu, has contributed to the articulation of diverse forms of Euroscepticism, ranging from moderate “Euro-realism” to radical calls for withdrawal from the EU. These actors mobilize identity-based narratives centered on nationalism, traditional values, and distrust of external influence.

At the same time, mainstream parties and civil society have responded by reaffirming Romania’s Western orientation, culminating in significant civic mobilization during the 2025 presidential elections. The findings suggest that, rather than rejecting European integration, Romanian society is increasingly negotiating the terms of its participation, seeking greater national autonomy within a pro-European framework.

## Introduction

The present country report was prepared as part of the EUPopLink COST Action ( Andreadis & Vasilopoulou, 2026) following the EUPopLink Theoretical Framework and guidelines ( Lorimer et al., 2026). Romania has generally exhibited a strong enthusiasm for EU membership, with public trust in the European Union consistently surpassing the European average. The latest Eurobarometer indicates that 56% of Romanians express confidence in the EU, a figure notably higher than the trust placed in national institutions (EB 102, 2024). There has been little overt opposition to the EU or the integration process from either the public or Romanian political figures. While acknowledging the potential for Euroscepticism, Mișcoiu (2021) notes that it remains in a nascent stage. Recently, the newly established populist party AUR (Alliance for the Union of Romanians) has tapped into underlying Eurosceptic sentiments within Romanian society, positioning itself as a significant opposition force. This rise of populist parties suggests that Euroscepticism is beginning to gain traction in Romania.

This evolving dynamic is further illuminated by data from a recent national survey ( INSCOP, 2025), which provides a revealing snapshot of where Romanian citizens stand on issues such as national sovereignty, economic independence, and trust in international partners like the EU, NATO, Russia, and China. Despite a widespread inclination to remain firmly embedded in Western structures, significant pockets of suspicion and cultural protectionism persist. To a more macro-analysis, an overwhelming majority of Romanian respondents prefer alliances with the West (EU, US, NATO) over the East (Russia, China), while also affirming Romania’s continued membership in the European Union and NATO). Indeed, more than 95% see the country’s proper alignment as Western; over 91% favour staying in the EU, and approximately the same share rejects leaving NATO.

However, to a more micro-analysis, this consensus for Euro-Atlantic partnerships coexists with a significant undercurrent of “sovereigntist” or anti-globalist sentiment, as indicated by the smaller though non-negligible percentages who either prefer an Eastern orientation or think leaving the EU and NATO might be warranted. Why this contradiction? Individuals voiced concerns about the EU’s impact on Romania’s national interests, the degree of foreign influence in the local economy, and the belief that the government acts at the behest of outside powers. Despite such scepticism toward Western institutions, the data show that most Romanians do not wish to sever ties altogether. Rather, they appear to hope for a more autonomous stance within these alliances – one in which Romania can assert national priorities while still reaping the security, economic, and political benefits of EU and NATO membership.

These tensions suggest that Romanians are not rejecting Western integration outright but are instead calling for a recalibration of Romania’s role within it. The prevailing desire appears to be for a more autonomous and assertive position within EU and NATO structures – one that safeguards national interests while still benefiting from the political, economic, and security advantages that come with membership. In this context, the rise of populist actors like AUR can be seen not only as a reaction against globalisation or external influence, but also as a demand for renegotiating Romania’s place in an increasingly complex international order.

## Populist political actors

Populism has been a persistent feature of post-communist Romanian politics, reaching its zenith in 2000 when Corneliu Vadim Tudor, leader of the prominent populist party, the Greater Romania Party (PRM), finished as runner-up in the presidential elections. However, from 2010 onwards, populism entered a period of dormancy, largely due to the reliance of populist parties on their leaders ( Corbu et al., 2016). By 2015, Vadim Tudor had passed away, and other populist leaders faced imprisonment for crimes such as blackmail and bribery. In this section, we provide an overview of the populist parties and actors currently active in Romanian politics.

However, the true turning point in the populist surge in Romania came in the aftermath of the 2019 European Parliament elections and the December 2020 parliamentary elections, when the previously unknown Alliance for the Union of Romanians ( *Alianța pentru Uniunea tuturor Românilor* – AUR) entered the national legislature with nearly 10% of the vote. AUR combined traditional nationalist rhetoric, anti-system populism, and conservative social values, appealing to both rural and urban voters disenchanted with the mainstream ( Crăciun & Țăranu, 2025). This momentum continued into the 2024 European Parliament elections, where AUR secured nearly 15% of the votes and six MEP seats. The party embodies characteristics typical of right-wing populism: nativism, authoritarianism, and populism ( Rooduijn et al., 2023). AUR’s platform centers on family, homeland, faith, and liberty, aiming to counteract traditional value erosion and defend national sovereignty against perceived EU threats ( Soare, 2024). Their rhetoric includes anti-elite sentiments, support for unification with Republic of Moldova (the “Greater Romania” narrative), opposition to EU and West-imposed gender ideologies and LGBTQ advocacy, and a revival of traditional family values rooted in Orthodox Christianity. George Simion, AUR’s president, is recognised as an effective communicator who actively engages with grassroots supporters. Simion entered the 2024 presidential race, finishing fourth in the first round. Following his strong criticism of the election annulment by the Constitutional Court, he ran again in the May 2025 presidential elections, this time securing first place in the first round. Ultimately, however, he was defeated in the runoff by the pro-EU independent candidate, Nicușor Dan.

Another populist party on the Romanian political landscape is S.O.S Romania ( *S.O.S România*, established in 2021 **).** The party’s dominant ideology, as the name suggests, revolves around the urgency of saving Romania ( Soare, 2024). The party is led by Diana Şoșoacă, a key figure of Romanian populism. Şoșoacă joined SOS Romania after being expelled from AUR, the party who propelled her to Romanian politics and offered her a senator seat. She is now a MEP and one of the most vocal populists in Bucharest and Brussels. Unlike AUR, SOS Romania seems to be totally dependent on the performance of its leader. SOS Romania displays nativist, authoritarian and populist dimensions and feature many of the rhetoric found in AUR. Mainly due to its leader verbal and symbolic violence and her antisemitism, SOS Romania is deemed a more extremist party than AUR. Şoșoacă’s candidacy for the 2024 presidential elections was invalidated by the Constitutional Court, a decision which generated uncontrolled and vitriolic outrage from her and was disapproved by the mainstream political parties and the Romanian society.

The newest populist party to enter Parliament following the December 2024 legislative elections, capitalising on the sovereigntist wave, is the Party of Young People ( *Partidul Oamenilor Tineri* – POT), established in 2023. The party is led by Anamaria Gavrilă, a former member of AUR. POT lacks a clear ideological platform and is primarily centered around its’ leader personality. Gavrilă’s discourse is marked by right-wing populist rhetoric, including anti-elitism, nationalism, and a strong appeal to traditional Orthodox Christian values. POT notably supported Călin Georgescu even before he gained public recognition or appeared in opinion polls as a presidential candidate. Following the annulment of the 2024 presidential elections, Gavrilă vocally endorsed Georgescu, condemning the Constitutional Court and later the Romanian Prosecutor’s Office, which charged Georgescu with serious offenses related to disturbing the constitutional order. Ahead of the 2025 presidential elections, Gavrilă and George Simion announced their candidacies strategically to prevent either’s disqualification by the Electoral Authority. Ultimately, Gavrilă withdrew her candidacy to back Simion. After Simion’s defeat, POT experienced significant internal turmoil, with numerous MPs accusing Gavrilă of intimidation. This discord led to the resignation of many senators and the subsequent dissolution of POT’s senatorial group. As political analyst Andrei Țăranu observed, unlike AUR, “POT is not a party, but more like a clique surrounding Anamaria Gavrilă” ( Digi24.ro, 2025).

Even more recently, the first round of the 2024 Romanian presidential elections came with a series of surprises that left many citizens, journalists, political analysts, and even political figures in a state of “bewilderment”. Latest to arrive among Romanian populists, Călin Georgescu managed to secure a frontrunner position at the 2024 presidential elections, generating a shock wave that has not yet been entirely processed by the Romanian society. The unknown, unexpected, TikTok-powered candidate imploded Romanian politics and triggered the election annulment by the Constitutional Court following suspicions of election meddling by Russia. Georgescu’s populism appeal on social media used simple nationalist rhetoric filled with religious overtones to effectively engage younger people and Romanian diaspora. Georgescu is a populist directly addressing the people, propagating conspiracy theories and presenting himself as the savior of Romania. POT and AUR, through their respective leaders, strongly supported Georgescu during the 2024 election campaign and in the aftermath of annulment. Following his prosecution and, potentially, the defeat of his protégé Simion in the 2025 presidential elections, Georgescu announced his retirement from political life. By early 2025, with Georgescu under investigation and politically sidelined, the populist mantle was passed to George Simion, the charismatic leader of AUR, who became the main contender in the re-run of the presidential elections. However, Simion’s performance in the 2025 campaign revealed the (current) limits of populist mobilisation. The victory of Nicușor Dan in the 2025 presidential elections signalled a temporary containment of the populist wave in Romania, but the electoral dynamics revealed that the base for Eurosceptic populism remains significant. Simion secured a robust second place, and the resonance of Georgescu’s narrative has left deep scars in the political imagination. While the institutions resisted the populist offensive in 2025, the political ecosystem has clearly changed.

## Positions on ‘Europe’ and the Populism-Euroscepticism nexus

Despite relatively low levels of popular Euroscepticism in Romania, party-based Euroscepticism is considerably more pronounced ( Taggart & Szczerbiak, 2002). Both forms appear to be driven by identity politics ( Stoica & Voina, 2023), which draw on historical, ethnic, and cultural dimensions of Romanian identity.

AUR, SOS Romania and Călin Georgescu all overtly express their Eurosceptic positions, although they diverge when it comes to the object of their opposition. While AUR displays an arguably ‘soft’ Euroscepticism, SOS Romania (via Diana Şoșoacă) and Călin Georgescu seem to be ‘hard’ Eurosceptic, promoting Romania’s exit from the EU that ruined the country and betrayed Romanians.

Claudiu Tîrziu, a former AUR senator and intellectual leader, declared that the party does not oppose the EU but the current EU policies that “torpedoed” the European project. Tîrziu rejects the label of “anti-European” for AUR, preferring to describe the party as “Euro-realistic” ( Agerpres, 2024). AUR has repeatedly criticized EU’s handling of the COVID pandemic (especially regarding vaccination), EU bureaucracy, EU’s intrusion into what they perceived to be the traditional, Christian values of the Romanian society. The party frames itself as a defender of these values against liberalizing trends promoted by Brussels, positioning cultural and religious identity at the core of its political message.

Following the success of the party in the 2024 EP elections, AUR sought to consolidate its position within the EP by making alliances with other parties sharing similar views. Gauging support for membership in the European Conservatives and Reformists, AUR intensified its networking efforts. Vox and Fratelli d’Italia supported AUR’s pledge and Simion participated in events organized by Vox or Meloni’s party. In April 2024, AUR organized in Bucharest an event called “Make Europe Great Again” (MEGA) aimed to critically assess Europe’s accentuating decline ( AUR, 2024). The second edition of MEGA was held in Brussels in January 2025. Launching this event series echoing Trump’s MAGA and participating to the recent inauguration of Trump’s second mandate are only two of examples of Simion’s appreciation of the US president. Accused of being a Russian proxy, Simion vehemently denied and tried to distance himself and his party from Russia. AUR condemned the Russian military aggression in Ukraine, a position which seemed to have been significantly influenced by the inclusion in the ECR group ( Soare, 2024).

AUR’s Euroscepticism is characterised by a critical but not outright rejectionist attitude toward the EU. The party challenges EU policies it views as harmful to national sovereignty, traditional values, and individual freedoms, while simultaneously seeking to reform the European project rather than dismantle it. Through strategic alliances and high-profile events, AUR aims to position itself as a key player within the European conservative-populist landscape, balancing nationalist rhetoric with pragmatic engagement in European institutions.

Unlike AUR, whose networking efforts paid off, SOS Romania and Diana Şoșoacă particularly failed in gaining support from other political groups in the EP. The two SOS Romania MEPs remained unaffiliated. Şoșoacă radically opposes the EU and the integration project, praising herself to be the first Romanian politician to advocate for a Ro-exit. This hardline Euroscepticism is not only rooted in nationalist rhetoric but also in a broader geopolitical stance that aligns closely with Russian interests. Şoșoacă has cultivated a close relationship with Russia, consistently amplifying Moscow’s anti-EU and anti-American narratives. Through her speeches and public appearances, she echoes Kremlin propaganda that portrays the EU as a coercive and illegitimate entity undermining national sovereignty. This alignment with Russian discourse further isolates her and SOS Romania from mainstream European political groups, reinforcing their position on the fringes of the EU political spectrum.

In addition to her Eurosceptic stance, Şoșoacă’s nationalist agenda extends to regional geopolitics. As a senator, she initiated legislation asserting Romania’s right to reclaim territories currently occupied by Ukraine, framing the conflict in terms of national sovereignty and territorial justice. This move underscores her broader rejection of the current European order and her willingness to challenge established international norms, reflecting a confrontational and uncompromising approach to both EU integration and regional diplomacy. Overall, SOS Romania’s Euroscepticism is characterised by a radical rejection of the EU, alignment with Russian geopolitical narratives, and a nationalist agenda that prioritizes sovereignty and territorial claims. This hard Euroscepticism contrasts sharply with more moderate or pragmatic positions within Romanian politics and contributes to the party’s political isolation both domestically and in the European Parliament.

Gavrilă’s POT holds a less clearly defined position on Europe compared to the parties previously discussed, and even less so than Călin Georgescu’s view. As the youngest populist party in Romania, lacking both ideological clarity and structural coherence, POT appears to have adopted the Eurosceptic sentiments of other populist parties and actors. Its endorsement of Călin Georgescu, a populist with strong Eurosceptic credentials, during the 2024 presidential elections further underscores its distance from pro-EU stances. While POT does not explicitly call for Romania’s exit from the EU, it is critical of deeper European integration and positions itself against perceived EU overreach. This stance aligns POT with other far right populist parties in Romania. The party leader’s rhetoric frames EU regulations and reforms as detrimental to Romanian interests, echoing broader populist narratives that portray Brussels as out of touch with the needs and values of ordinary Romanians.

Overall, POT’s platform is marked by a blend of Euroscepticism and nationalism, using anti-elite, sovereigntist, and traditionalist rhetoric to appeal to voters dissatisfied with both the EU and Romania’s political establishment.

Călin Georgescu’s ‘hard’ Euroscepticism is exemplified by his declarations regarding removing Romania from EU and NATO once he becomes president. More striking and troublesome are his recently affirmations about Ukraine being an “invented state” whose dissolution Romania should benefit from ( România Liberă, 2025). This triggered a quick reaction from the Romanian Foreign Affairs Minister who reconfirmed the country’s support for Ukraine’s independence and territorial integrity.

## Responses to Euroscepticism and consequences

The rise of populist parties in Romania has been accompanied by a marked intensification of Eurosceptic discourse, challenging the traditional pro-European consensus. In response, mainstream political parties have increasingly co-opted elements of populist rhetoric, including nationalist and sovereigntist themes, to broaden their electoral appeal and counter the growing influence of populist movements ( Corbu et al., 2016). Major pro-European parties, however, have generally avoided direct, confrontational challenges to Eurosceptic views. Instead, they prioritised highlighting the tangible benefits of EU membership, sidestepping deeper engagement with the identity-based grievances that underpin Euroscepticism.

The annulment of the 2024 presidential elections can be interpreted as a political reaction to the surge in populism and Euroscepticism, signalling institutional resistance to what was perceived as a destabilizing challenge to the established order. Nonetheless, the effectiveness of this measure in raising public awareness about the tactics and implications of populist actors remains uncertain. While the annulment may have temporarily disrupted populist momentum, it also risked being perceived as an undemocratic intervention, potentially reinforcing populist narratives about elite manipulation and undermining trust in democratic institutions.

In the May 2025 presidential elections, Romania witnessed a remarkable mobilisation of civil society actors who played a crucial role in countering the surge of populism and Euroscepticism. Following the annulment of the 2024 elections amid concerns of foreign interference favouring far right nationalist candidates, civic groups, independent media, and pro-democracy organisations intensified their efforts to raise public awareness about the dangers posed by populist and Eurosceptic forces. A massive popular mobilisation took place between the two rounds of the elections, culminating on Europe’s Day (May 9, 2025) when citizens gathered in the street to reaffirm Romania’s democratic values and its commitment to a European and Western future ( Hotnews.ro, 2025). The election of Nicușor Dan, a former civic activist and pro-European independent candidate, symbolised the culmination of these efforts, marking a significant victory for democratic resilience and the pro-Western consensus in Romania.

## Short conclusion

In conclusion, the current political direction in Romania highlights a visible shift between rising populism and Euroscepticism, alongside persistent efforts by mainstream parties and civil society to uphold democratic and pro-European values. Populist parties such as AUR, POT, and SOS Romania have capitalised on nationalist sentiments, anti-elitist rhetoric, and scepticism toward the European Union, challenging Romania’s traditional pro-European consensus. While their approaches vary, from AUR’s “Euro-realistic” stance to the hardline Euroscepticism of SOS Romania and the more ambiguous position of POT, these movements collectively amplify concerns about national sovereignty, cultural identity, and perceived EU overreach. At the same time, mainstream political forces and civil society actors continue to defend democratic standards and the benefits of European integration. The coming years will be crucial in determining whether Romania will maintain its path within the European mainstream or move further toward a politics shaped by division, distrust, and nationalist rhetoric.

## Ethics and consent

Ethical approval and consent were not required.

## Data Availability

No primary data were collected or analysed for this study. The article is based exclusively on publicly available reports and sources, which are fully cited in the reference list.
