# Supplementary material for: EUPopLink Country report - Romania
Source: Open Res Eur. 2026 May 14;6:167. [Version 1] doi: 10.12688/openreseurope.23593.1 (PMC13234545; doi:10.12688/openreseurope.23593.1)
Supplement: Peer review history

# Reviewer report: Reviewer response for version 1

**DOI:** 10.21956/openreseurope.25541.r74564

**Published:** 2026-06-03

**Authors:** Piotr Zagórski

**Affiliations:** 1 Institute of Philosophy and Sociology Polish Academy of Sciences, Warsaw, Masovian Voivodeship, Poland

This country report provides a highly commendable, timely, and sophisticated analysis of the Romanian political landscape following the turbulent 2024–2025 electoral cycle. The authors successfully map the rapidly shifting boundaries between "soft" and "hard" Euroscepticism using established frameworks, providing vital clarity on how reformist actors like AUR differ from more radical, pro-Russian figures like Diana Șoșoacă and Călin Georgescu. Capturing the historic Constitutional Court annulment and the subsequent May 2025 presidential re-run while maintaining academic objectivity is a significant achievement, making this an engaging, analytically sharp, and highly relevant contribution to the EUPopLink COST Action.

 Nonetheless, a few key conceptual and theoretical refinements could be made to further enrich the report. First, it overlooks a major structural shift in Romanian politics: neither of the traditional establishment giants, the Social Democratic Party (PSD) or the National Liberal Party (PNL), even qualified for the May 2025 presidential runoff. The analysis would benefit from explaining how this institutional decay created the vacuum that allowed an independent centrist and a right-wing populist to dominate the final vote, as the growth of Euroscepticism seems structurally tied to the failure of the mainstream establishment.

 Second, the introduction attempts to frame overall institutional alignment as a "macro-analysis" and policy-specific anxieties as a "micro-analysis." In political science, macro/micro indicators usually signify distinct levels of analysis (e.g., system-level structures vs. individual-level behaviors). Because both sets of findings in this report originate from individual-level survey responses (INSCOP and Eurobarometer), this terminology is conceptually confusing. Inclusion of a footnote about basic data (sample, date, methodology etc.) of the “INSCOP: Public Attitudes Toward Economic Patriotism and the Vulnerabilities of the Democratic Regime” survey would also be helpful. 

 Third, the authors note that Romanian society is "seeking greater national autonomy within a pro-European framework" and calling for a "more autonomous and assertive position within EU and NATO structures" that "safeguards national interests while still benefiting from... membership." While this description captures public sentiment well, it remains conceptually somewhat vague. It would help if the authors clarify what this ambiguous positioning means in practice. Are voters and moderate populist actors advocating for a reformist, anti-federalist vision of the EU or are they shifting toward a purely transactional, instrumental relationship with Western alliances?

 Finally, it would be interesting to know the reasons for why “SOS Romania and Diana Şoșoacă particularly failed in gaining support from other political groups in the EP”, as ideologically they would probably be a quite close fit to the ESN group. This exclusion demonstrates that there are boundaries of acceptability operating even within the most radical fringe of the EP.

Is the case presented with sufficient detail to be useful for teaching or other practitioners?

Yes

Is the work clearly and accurately presented and does it cite the current literature?

Yes

If applicable, is the statistical analysis and its interpretation appropriate?

Not applicable

Are all the source data underlying the results available to ensure full reproducibility?

No source data required

Are the conclusions drawn adequately supported by the results?

Yes

Is the background of the case’s history and progression described in sufficient detail?

Yes

Reviewer Expertise:

Electoral behavior, populism, Central and Eastern Europe

I confirm that I have read this submission and believe that I have an appropriate level of expertise to confirm that it is of an acceptable scientific standard.

# Reviewer report: Reviewer response for version 1

**DOI:** 10.21956/openreseurope.25541.r74565

**Published:** 2026-06-01

**Authors:** Sorina Soare

**Affiliations:** 1 University of Florence, Firenze, Italy

 It is a very solid report,  balanced, concise, and to the point. I would suggest a few small clarifications and additions only to increase the readability for non-experts on Romania.

 It may be useful to clarify the invalidation of Șoșoacă’s candidacy and better distinguish it from Georgescu’s case, particularly regarding the legal and political rationale behind the different treatments.

 A brief nuance could also be added regarding Georgescu's retirement from politics, especially in relation to his continued public visibility and participation in debates.

 It would also help to include a couple of lines on the limited solidity of SOS and POT as parliamentary groups, as well as on the formation of PACE.

 In addition, a short comment on the recent motion and the partial dismantling of the cordon sanitaire would strengthen the analysis, particularly in light of the growing fragmentation within parliament.

 I would also suggest adding a comparative table clarifying the differences between AUR, SOS, and POT about Euroscepticism. More broadly, the report could further clarify the parties’ respective positions toward the EU project, including which EU policies they contest most strongly; their preferred level of interaction with the United States versus the EU; openness toward alternative geopolitical alignments, including China and Russia; and possibly their positioning on Ukraine as part of this broader framework. Overall, these additions would further strengthen an already very solid and well-balanced report.

Is the case presented with sufficient detail to be useful for teaching or other practitioners?

Yes

Is the work clearly and accurately presented and does it cite the current literature?

Yes

If applicable, is the statistical analysis and its interpretation appropriate?

Not applicable

Are all the source data underlying the results available to ensure full reproducibility?

Yes

Are the conclusions drawn adequately supported by the results?

Yes

Is the background of the case’s history and progression described in sufficient detail?

Yes

Reviewer Expertise:

populism, party politics, diaspora politics

I confirm that I have read this submission and believe that I have an appropriate level of expertise to confirm that it is of an acceptable scientific standard.
